# Role of Slit/Robo Signaling pathway in Bone Metabolism

**DOI:** 10.7150/ijbs.66931

**Published:** 2022-01-09

**Authors:** Lingyu Jiang, Jianxun Sun, Dingming Huang

**Affiliations:** 1 State Key Laboratory of Oral Diseases and National Clinical Research Center for Oral Diseases, West China Hospital of Stomatology, Sichuan University, Chengdu 610041, China; 2 Department of Conservative Dentistry and Endodontics, West China Hospital of Stomatology, Sichuan University, Chengdu 610041, China

**Keywords:** Slit/Robo, Coupling factors, Bone Metabolism, Osteoblast, Osteoclast

## Abstract

Slit/Robo signals were initially found to play an essential role in nerve development as axonal guidance molecules. In recent years, with in-depth study, the role of Slit/Robo in other life activities, such as tumor development, angiogenesis, cell migration, and bone homeostasis, has gradually been revealed. Bone is an organ with an active metabolism. Bone resorption and bone formation are closely related through precise spatiotemporal coordination. There is much evidence that slit, as a new bone coupling factor, can regulate bone formation and resorption. For example, Slit3 can promote bone formation and inhibit bone resorption through Robo receptors, which has excellent therapeutic potential in metabolic bone diseases. Although the conclusions of some studies are contradictory, they all affirm the vital role of Slit/Robo signaling in regulating bone metabolism. This paper reviews the research progress of Slit/Robo signaling in bone metabolism, briefly discusses the contradictions in the existing research, and puts forward the research direction of Slit/Robo in the field of bone metabolism in the future.

## Introduction

**Figure 1:**
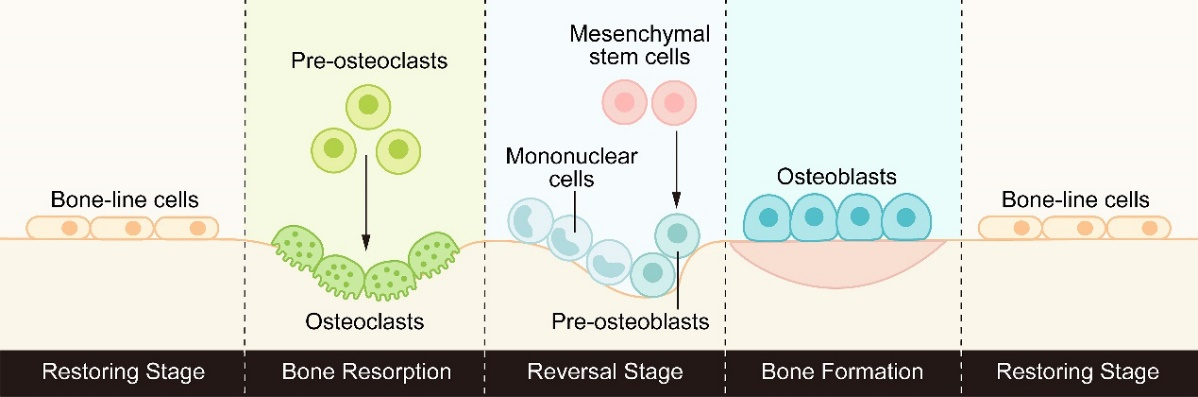
The process of bone remodeling: osteoclasts initiate bone resorption, then the reverse stage begins under the activation of signal molecules, transforming from bone catabolism to bone synthesis metabolism, and finally osteoblasts form new bone to replace old bone.

Bone is a highly dynamic tissue and organ that goes through the process of bone formation and bone resorption throughout life to maintain good mechanical properties and maintain the steady state of minerals in the tissue. This process of continuous replacement is called bone remodeling 1. Bone remodeling usually consists of three consecutive stages: osteoclasts initiate bone resorption, a transition from osteoclast-dominated catabolism to osteoblast-dominated anabolism, and finally osteoblasts form the bone matrix and replace old bone with new bone. (Figure 1).

In healthy bone, each stage of bone remodeling is subject to precise spatiotemporal regulation to ensure that the absorbed bone is equal to the newly formed bone, maintaining a balance between osteoclasts and osteoblasts. This coordinated relationship between bone resorption and bone formation in time and space is called bone coupling 2, 3. Since this concept was proposed, the subtle interaction between osteoclasts and osteoblasts has been a hot topic. Understanding the mechanism of bone coupling has important guiding significance for the treatment of metabolic bone diseases caused by bone resorption or steady-state destruction of bone formation, such as osteoporosis and ossification 4.

The growth, development, and repair of bones depend on correct innervation. Neuronal axons and osteoblasts form membrane-membrane contacts, which mediate direct communication between cells through molecules such as glutamate and substance P^5^-^7^. The physical and functional connection between the nervous system and the skeletal system forms a neuroskeletal network 8. To date, it has been reported that BMPs 9, 10, CGRP 11, 12, CXCR4/CXCL12 13, 14, Eph/ephrin 15, 16, and other molecules mediate the crosstalk between bone and the nervous system and participate in the formation of the neuroskeletal network.

In recent years, an increasing number of studies have found that axon guiding molecules such as Sema 17, Netrins 18, 19, and Slit 20, 21 are also members of the neuroskeletal network. They play an essential role in neural development and are expressed in the bone microenvironment to mediate the interaction between osteoblasts and osteoclasts. Sema3A can inhibit the differentiation of osteoclasts by suppressing RhoA and enhance the differentiation of osteoblasts mediated by β-catenin 22. At the same time, Netrin-1 can promote the activation of RhoA in osteoclasts via UNC5B receptors, which promotes the fusion of osteoclasts and antagonizes the role of Sema3A in the bone microenvironment 23, 24. The Slit family controls axonal rejection and axonal guidance by binding to Robo receptors, which are essential in nerve development 25. With the in-depth study of this family, it was found that they are also involved in the regulation of cancer development, cell migration, cell proliferation, and angiogenesis and play an essential role in bone metabolism 26-28. This article reviews the research progress and future research direction of the Slit/Robo signaling pathway in bone metabolism.

## Slit Ligands and Robo Receptors

### The Structure and Proteolysis of Slit Ligands

Slit family proteins are highly conserved secretory proteins that were first found in the central nervous system of Drosophila embryos. They are synthesized by midline glial cells and control midline crossover, axon rejection, and axon guidance 29. Since then, Slit homologs have been found from nematodes to fish, birds, amphibians, and mammals, which are highly conserved among species. There is only one type of slit (Slit1) in invertebrates, while there are three *slit* genes (*Slit1~3*) in most vertebrates, which encode approximately 200 kDa 26. The *Slit1* gene is located on human chromosome 10q24.1, and the *Slit2* gene is located on 4p15.31. The *Slit3* gene is located on 5q34-q35.1 30.

**Figure 2:**
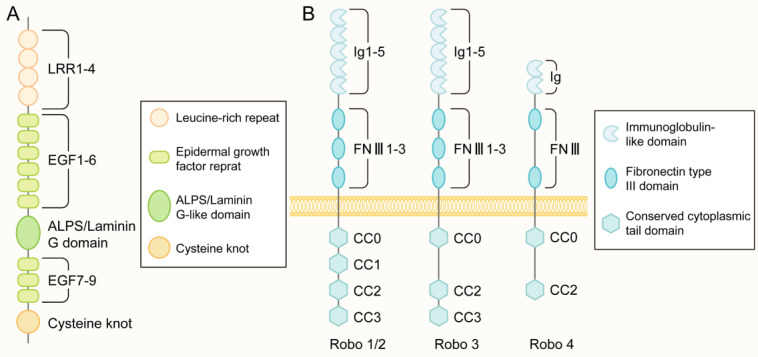
** The structure of Slit and Robo.** (A) The molecular structure of the slit ligand, from the N-terminus to the C-terminus, is mainly composed of five structural parts: four leucine-rich repeats (leucine-rich repeats, LRRs), six epidermal growth factor (epidermal growth factor, EGF) repeats, an ALPS domain or laminin-G-like domain, another three EGF repeats and one cysteine junction. (B) The molecular structure of the Robo receptor protein: Robo1-3 has five Ig-like domains, three fibronectin type III repeat sequences, one transmembrane part, and a long cytoplasmic tail. The cytoplasmic tail of Robo1/2 contains four conserved proline-rich cytoplasmic motifs, called CC0-CC3. In contrast, the cytoplasmic tail of Robo3 has CC0, CC2, and CC3 domains. There are only two Ig-like domains and two FNIII domains in the extracellular part of Robo4 and only CC0 and CC2 domains in the intracellular region.

The protein encoded by the *Slit* gene mainly consists of five structural parts from the N-terminus to the C-terminus: four leucine-rich repeats (LRRs), six epidermal growth factor (EGF) repeat sequences, and a domain found in agrin, laminin, perlecan, and slit named the ALPS or laminin-G-like domain. In addition, there were one to three EGF repeats and one cysteine knot (Figure 2A). The main difference in Slit1-3 lies in the number of EGF repeat sequences. Slit protein can form a homodimer through the reaction between the LRR2 domain and the LRR4 domain 31. In addition, Slit can bind to other molecules in the extracellular matrix, including Type IV Collagen 32, Netrin 1^33^ and dystroglan 34.

**Figure 3:**
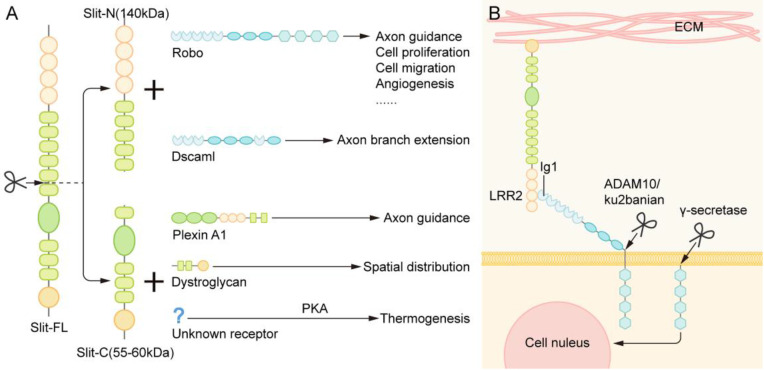
** The Proteolysis of Slit and Robo.** (A) The slit protein has a conserved proteolysis site between the fifth and sixth EGF repeats and is cleaved by an unknown protease to grow the N-terminal fragment (Slit-N) and short C-terminal piece (Slit-C). They can bind to different receptors to perform various functions. (B) The binding of the Slit protein fixed to the extracellular matrix to the Robo on the cell membrane results in the exposure of the cleavage site of metalloproteinases in the area adjacent to the membrane of Robo protein. Under the action of Kuzbanian in Drosophila (ADAM10 in mammals), the Robo protein is cleaved, and its extracellular domain falls away. In human cancer cells, the lagged residues of the extracellular domain of Robo are further cleaved by γ secretase, and the resulting C-terminal fragments can then be transferred to the nucleus.

The slit protein has a conserved proteolysis site between the fifth and sixth EGF repeats and can be cleaved by an unknown protease to grow an N-terminal fragment (Slit-N) and short C-terminal fragment (Slit-C) 35. These different forms of Slit proteins have different functions (Figure 3A).

### Receptors of Slit Ligands

Robo family proteins are the main receptors of Slit ligands. In the majority of tissue microenvironments, they combine with each other to transmit cellular signals. Both full-length Slit (Slit-FL) and Slit-N can bind to Robo receptors and regulate axonal guidance, cell proliferation, cell migration, angiogenesis, and other life activities 26. However, the interaction between Slit and Robo is not unique. Several novel receptors of Slit have been found in recent studies.

Dan et al. 36 found that Slit-N can directly bind to the N-terminal Ig domains of Dscam1, which enhances the interaction between Dscam1 and receptor tyrosine phosphatase 69D (RPTP69D) and stimulates the dephosphorylation of Dscam1. It does not depend on Robo to promote the extension of specific axon collaterals.

Slit-C is mainly released into the extracellular space. PlexinA1, a novel receptor of Slit, can bind to the C-terminal fragment of Slit specifically and mediate commissural growth cone collapse independently of the Robo. Slit/PlexinA1 and Slit/Robo signaling play a complementary role in the guidance of commissural axons 37.

In addition, Kevin et al. 38 found that the C-terminus of Slit can bind to the floor plate and basement membrane scaffold protein Dystroglycan to regulate the spatial distribution of cells. In mutant mice with dystroylycan deletion, the distribution of endogenous Slit was wrong and led to developmental abnormalities. The combination of Slit and Dystroglycan is an important factor affecting the correct distribution of Slit *in vivo*.

A recent study 39 has shown that the C-terminal fragment of Slit2 can activate PKA-dependent signaling pathways to regulate adipose tissue thermogenesis and improve blood glucose homeostasis, which shows therapeutic potential for the treatment of obesity. However, the exact receptor of Slit2-C in this signaling pathway remains unclear.

### The Structure and Proteolysis of Robo Receptors

The most important receptor of the Slit family is the Robo protein, which is a type of transmembrane receptor protein composed of 1000 to 1600 amino acids that has a highly conserved intracellular domain. There are three Robo receptors (Robo1-3) in Drosophila, chickens, and Xenopus laevis and four Robo receptors (Robo1-4) in zebrafish and mammals 40, 41. The *Robo1* and *Robo2* genes are located on human chromosome 3p12.3, and the *Robo3* and *Robo4* genes are located on human chromosome 11q24.2 42.

The Robo family is a family of immunoglobulin (immunoglobulin, Ig) superfamilies. Robo1-3 has five Ig-like domains, three fibronectin type III repeats, a transmembrane part, and a long cytoplasmic tail (Figure 2B). The cytoplasmic tail of Robo1 and Robo2 contains four conserved proline-rich cytoplasmic motifs, while the cytoplasmic tail called CC0-CC3; Robo3 contains CC0, CC2, and CC3 domains. Robo4 was initially considered a vascular endothelial cell-specific receptor of the Robo family^41^, with only two Ig-like domains and two FNⅢ domains in the extracellular part and only CC0 and CC2 domains in the intracellular part(Figure 2B). A recent study 43 showed that Robo4 also exists in the newborn cerebral cortex to regulate the directional migration of newborn neurons. No apparent catalytic region was found in the Robo family proteins, mainly through the CC0-CC3 domain in the cytoplasm to bind to various intracellular signal molecules to mediate signal transmission.

Robo proteins can also be cleaved, which seems to be essential for the signal transduction of the Slit/Robo pathway into the cell. The binding of the Slit protein, which is fixed to the extracellular matrix, to the Robo on the cell membrane results in the exposure of the cleavage site of metalloproteinases in the area adjacent to the membrane of Robo protein. Under the action of Kuzbanian in Drosophila (ADAM10 in mammals), the Robo protein is cleaved, and its extracellular domain falls away 44, 45 (Figure 3B). Some studies have found that the cleavage of Robo receptors is necessary to recruit downstream signaling molecules. In human cancer cells, the residue of the extracellular domain of Robo is further cleaved by γ secretase, and the resulting C-terminal fragment can then be transferred to the nucleus. However, the specific function of this fragment has not been determined 45 (Figure 3B). Nevertheless, it remains unclear whether the Robo of all vertebrates will exfoliate extracellular domains and play a role in the development.

### Ligands of Robo Receptors

Slit protein is considered the only ligand of Robo receptors. The second leucine-rich repeat (LRRD2) of the Slit protein is its active site, which binds to the active site in the first Ig domain of Robo1 and Robo2, thus transducing the extracellular signal into the cell. Robo3 and Robo4 are two unique receptors in the Robo family. Due to some amino acid changes in its first Ig domain, Robo3 almost lost its ability to bind Slit protein 46. However, Robo3 can acquire new signaling properties simultaneously, such as NELL ligands.

NELL family proteins are newly discovered ligand proteins of Robo2/3 and cannot bind to Robo1/4. However, because the extracellular hairpin-like domain of Robo2 may mask the binding site of NELL1/2, NELL1/2 can bind to Robo2 only after the conformational change of the extracellular domain of Robo2 under mild acidic conditions 47, 48. Both NELL1 and 2 can bind to Robo3. NELL2 can antagonize the rejection induced by Slit2-Robo1/2 and allow the commissural axon to cross the midline by binding to the Robo3 receptor 49. Although NELL1 does not play an important role in this, it may become an effective ligand of Robo3 in other life activities 50.

Robo4 is believed to be specifically expressed in endothelial cells 28, and thus far, there have been no reports of direct interaction between Robo4 and Slit2/3. Some researchers 51-53 have proposed that Robo4 transmits signals by forming coreceptors with other molecules, such as Robo1 and heparan sulfate proteoglycans (HSPGs). The current research on Robo3 and Robo4 is imperfect. Whether they bind to Slit ligands and regulate cell activity remains to be determined.

The relationship between Slit/Robo signaling and other relevant ligands or receptors forms a complex regulatory network, which not only increases its richness and diversity but also provides a sufficient theoretical basis for its role in many life activities.

### Slit-Robo Signaling in Bone Metabolism

Bone remodeling is performed by a structure called basic multicellular units (BMUs), which are mainly composed of two types of cells: osteoblasts and osteoclasts 54, 55. The whole stage of bone remodeling is composed of catabolism of osteoclasts and synthetic metabolism of osteoblasts: it begins with the activation of bone resorption of osteoclasts. After the completion of the resorption stage, preosteoblasts are recruited to the bone surface, differentiate and mature, begin the synthesis of bone matrix, and form new bone to replace old bone. The signaling molecules that coordinate the bone resorption of osteoclasts and the bone formation of osteoblasts in BMUs are called bone coupling factors. The slit protein family, the classical axon guide molecule, is a recently discovered bone coupling factor that plays a vital role in regulating bone metabolism 2. The expression of Slit1 is the lowest in bone tissue, while Slit2 and Slit3 are expressed in both osteoblasts and osteoclasts, although the expression level may vary according to the state of cells 56, 57. Robo1 and Robo3 are expressed in osteoclasts, Robo1 and Robo2 are expressed in osteoblasts, and Robo4 is hardly expressed in osteoblasts 58. In recent years, there have been an increasing number of studies on the role of Slit2 and Slit3 in bone metabolism, and the regulatory role of Slit2 and Slit3 in osteoblasts and osteoclasts is gradually revealed.

### The Effect of Slit2 on osteoblasts and osteoclasts

**Figure 4:**
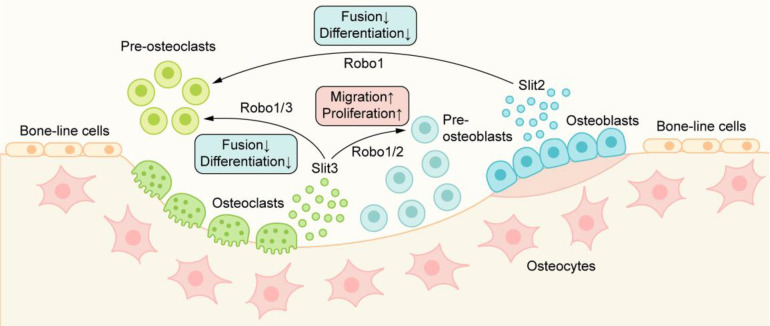
The role of Slit/Robo signaling in metabolic bone coupling: Slit2/3 acts on osteoclasts through the Robo1/3 receptor to inhibit the migration and fusion of osteoclasts, resulting in the inhibition of osteoclast formation. Slit3 can also bind to the Robo1/2 receptor on osteoblasts and promote the migration and proliferation of osteoblast lines, thus promoting bone formation.

The occurrence of osteoclasts is mainly carried out through the proliferation, migration, and fusion of preosteoclasts and the subsequent differentiation of osteoclasts. Slit2 plays a role in the early stage of osteoclast development, which is directly related to the Robo1 receptor on preosteoclasts and inhibits osteoclast differentiation 59 (Figure 4). In the presence of Slit2, the expression of osteoclast surface differentiation markers, such as tartrate-resistant acid phosphatase (TRAP) and calcitonin receptor (CTR), decreased significantly. Slit2 also inhibited the activity of the small GTPase Cdc42 but had no significant effect on the activity of Rac and RhoA, resulting in a significant decrease in the migration and fusion of preosteoclast cells and resulting in the inhibition of osteoclast formation 59.

A previous study 56 pointed out that Slit2 inhibited the differentiation of osteoblast lines mainly through Robo receptors and inhibited the alkaline phosphatase activity of rat osteoblasts and MC3T3 cell lines in a concentration-dependent manner. Furthermore, Slit2 changed the cell morphology from flattened to elongated but had no significant effect on the proliferation of osteoblasts. However, a recent study 59 showed that the treatment of osteoblasts with recombinant Slit2 could not change the activity, migration ability, osteoblast differentiation markers, or alkaline phosphatase activity of osteoblast lines. The reason for this contradictory result may be that the conditioned medium for the treatment of osteoblasts in the previous study contains not only inexactly identified Slit2 but also other molecules that affect the biology of osteoblasts. In addition, cell type, cell microenvironment, treatment time, treatment conditions, and other factors may affect the effectiveness of the Slit/Robo pathway, which may also be one of the explanations for the contradictory phenomena in different studies.

### The Effect of Slit3 on osteoblasts and osteoclasts

In recent years, Slit3 has been shown to be a new bone coupling factor. Kim et al. 58 found that Slit3 derived from osteoclasts acts on osteoblasts in the form of paracrine signaling to promote bone formation and acts on osteoclasts in the form of autocrine signaling to inhibit bone resorption(Figure 4). Slit3 interacts with Robo1 and Robo3 receptors on osteoclasts/preosteoclasts, which inhibits the small GTPase Rac1. In addition, Slit3 also reduces the expression of DC-STAMP in preosteoclasts, and DC-STAMP plays an important role in the fusion of preosteoclasts. Slit3 can inhibit the formation of osteoclasts and the bone resorption of osteoclasts by mediating the disturbance of migration and fusion of preosteoclasts and reducing the expression of osteoclast differentiation markers such as TRAP and CTR, but Slit3 does not affect the proliferation of preosteoclasts 58. In osteoblasts, Slit3 binds to Robo1 and Robo2 receptors on the cell membrane, activates β-catenin, stimulates the migration and proliferation of osteoblast lines in a concentration-dependent manner, and promotes bone formation 27, 58. In the mice with osteoclast-specific deletion of Slit3, the bone formation parameters decreased while the bone resorption parameters increased, which showed a decrease in bone mass. In contrast, bone mass was normal in mice with osteoblast-specific deletion of Slit3 or neuron-specific deletion of normal 58. These results suggest that osteoclast-derived Slit3 may be a stop signal of bone resorption during bone remodeling and initiate the reversal stage of osteoblast bone formation.

However, Xu et al.^60^ found that Slit3 was strongly expressed in osteoblasts but hardly expressed in osteoclasts; similarly, osteoblast-specific SLIT3-deficient mice showed a decrease in bone mass, while osteoclast-specific Slit3-deficient mice showed a normal bone mass, and they believed that osteoblasts were the primary source of Slit3 in bone. The results of Li et al. 61 also supported this finding that there was no significant effect on the number, differentiation, bone resorption/bone formation parameters, or total bone mass of osteoclasts in the absence of Slit3 in osteoclasts.

We suspect that the differences in the above experimental results may be partly due to the differences in tissue-specific clones used to produce conditional gene knockout mice or the existence of other unknown compensatory mechanisms in osteoclasts. Although the primary source of Slit3 in bone and its role in osteocytes are still under debate, it is certain that Slit3 plays an essential role as a bone coupling factor in bone metabolism, and further research is needed to better understand and clarify the role of Slit3-Robo signaling in bone metabolism.

### Downstream of Slit-Robo Signaling in Bone Metabolism

**Figure 5:**
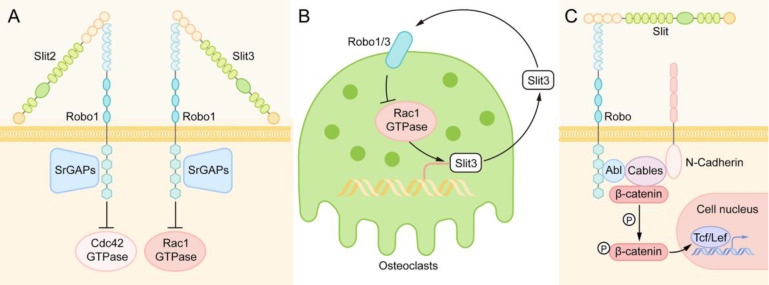
** Downstream of Slit-Robo Signaling in Bone Metabolism.** (A) When Slit binds to Robo, Slit-Robo GTPase activating protein (srGAP) is recruited to the intracellular domain of the Robo receptor, resulting in inactivation of Rho GTPase, which inhibits actin polymerization and stress fiber formation and affects cell polarity and cell movement. Slit2 mainly inhibits Cdc42 GTPase, while Slit3 mainly inhibits Rac1 GTPase. (B) In osteoclasts, activated Rac1 can stimulate the expression of Slit3. Slit3 acts on osteoclasts in an autocrine way, binds with Robo1 and recruits srGAP2 to form a complex, and inhibits the activation of Rac1 to form a negative feedback loop. (C) After binding to the Slit ligand, the Robo protein forms a multimolecular complex with Abl kinase, adaptor protein cables, N-cadherin and β-catenin. Then, Abl kinase phosphorylates β-catenin on tyrosine 489, resulting in the release of N-cadherin-related β-catenin, leading to the loss of N-cadherin-mediated cell adhesion. The released β-catenin can also enter the nucleus to activate target genes and regulate the life activities of cells.

Small GTPases of the Rho family, including RhoA, Cdc42, and Rac1, play an essential role in the downstream reaction of Slit/Robo signaling. Osteoclasts are responsible for reorganizing the cytoskeleton, which is essential for cell movement, and promoting the occurrence and development of specialized structures needed for bone resorption 62, 63. After the activation of the Slit/Robo signaling pathway, Slit-Robo GTPase activating proteins (srGAPs) are recruited to the intracellular domain of the Robo receptor, resulting in the inactivation of Rho GTPase, thus inhibiting cell actin polymerization and stress fiber formation and affecting cell polarity and cell movement 42, 64 (Figure 5A). However, different Slit family members have different inhibition of the Rho GTPase. Slit2 mainly inhibits the Cdc42 GTPase 59, while Slit3 mainly inhibits the Rac1 GTPase 58. There is also a feedback loop in osteoclasts. Activated Rac1 can stimulate an increase in Slit3 expression. Slit3 acts on osteoclasts in an autocrine way, forms a complex with Robo1 and recruits srGAP2, and inhibits the activation of Rac1 64 (Figure 5B).

In addition to the Rho GTPase family, another major signaling molecule downstream of Robo receptors is cytoplasmic kinases, in which Abelson (Abl) tyrosine kinase is the main participant 26. After binding to the Slit ligand, the Robo protein forms a multimolecular complex with Abl kinase, adaptor protein cables (Cdk5 and Abl enzyme substrate), N-cadherin, and β-catenin. Then, Abl kinase phosphorylates β-catenin on tyrosine 489, resulting in the release of N-cadherin-related β-catenin, which leads to the loss of N-cadherin-mediated cell adhesion 65, 66. The released β-catenin can also enter the nucleus to activate target genes and regulate the life activities of cells 27 (Figure 5C).

Wnt/β-catenin plays an important role in the control of bone development. Slit3 has been shown to promote chondrocyte differentiation and regulate endochondral ossification by binding to the Robo2 receptor to inhibit the activity of β-catenin in chondrocytes 67, which is contrary to its effect on osteoblasts. Slit3 binds to the Robo1/2 receptor to activate β-catenin in osteoblasts to stimulate osteoblast proliferation and migration 58. The different effects of Slit3 on the activity of β-catenin are related to the cell type. Moreover, we speculate that it is one of the reasons that different regions of the Abl tyrosine kinase have different effects on Robo receptors. Some studies 26, 66 have found that Abl kinase can inhibit Robo signaling by phosphorylating the CC1 domain of Robo, and it can also promote Robo signaling by binding to Capulet protein or Cables protein. However, in different cells, the specific mechanism of regulating the effect of Abl kinase on Robo signaling has not been reported clearly.

### The Interaction of Slit-Robo Signaling in Angiogenesis and Bone Metabolism

In the skeletal system of mammals, angiogenesis plays an essential role in maintaining the dynamic balance of bone remodeling. Blood vessels can not only provide necessary nutrients, oxygen, growth factors, and hormones for bone tissue but also secrete signaling molecules to regulate bone formation 68, 69. This close relationship between angiogenesis and bone formation in time and space is called "angiogenesis-osteogenesis coupling. " 70

**Figure 6:**
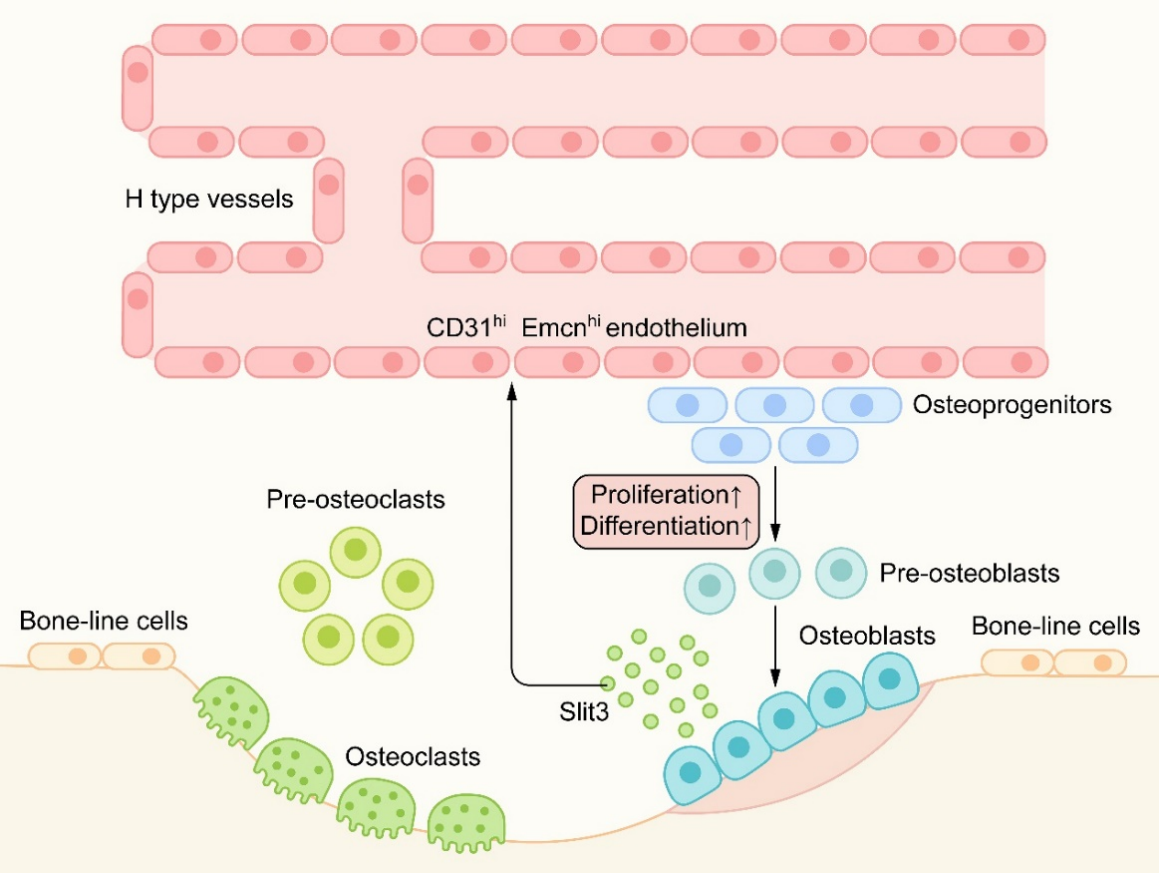
Slit/Robo in angiogenesis-osteogenesis coupling: H-type blood vessels are surrounded by dense Osterix+ bone progenitor cells. H-type blood vessels can guide bone formation by stimulating the proliferation and differentiation of bone progenitor cells. Osteoblast-derived Slit3, as an angiogenic factor, can produce an increase in CD31hiEmcnhi endothelium. Hence, Slit3 indirectly promotes bone formation and couples the process of bone metabolism with angiogenesis.

In recent studies 60, a capillary subtype, called H-type blood vessels, was significantly related to osteogenesis, which is characterized by the high expression of CD31 and Emcn on endothelial cells. H-type blood vessels are mainly located in the metaphysis and surrounded by dense Osterix+ bone progenitor cells. H-type blood vessels can guide bone formation by producing factors that stimulate the proliferation and differentiation of bone progenitor cells 71 (Figure 6).

Robo1, Robo2, and Robo4 are expressed in all types of vascular endothelial cells. Slit2/3 can regulate angiogenesis by combining with the Robo1 or Robo1/Robo4 heterodimer 42, 51. Osteoblast-derived Slit3 has been shown to increase the number of H-type vascular endothelial cells, couple the process of bone formation with angiogenesis, and indirectly promote bone formation as a pro-angiogenic factor 72 (Figure 6). Mice lacking Slit3 or Robo1 showed a significant decrease in H-type endothelial cells and bone mass. In a mouse fracture model, recombinant Slit3 significantly increased the number of H-type blood vessels and promoted fracture healing 60. These results suggest that Slit3/Robo1 is a strong regulatory signal for inducing H-type angiogenesis and promoting bone formation and plays an important role in the angiogenesis-osteogenesis coupling.

Interestingly, some studies 42 have shown that Slit/Robo signaling can not only promote angiogenesis through some receptors but also inhibit angiogenesis through other receptors. For example, Slit2/Robo1 can promote tumor angiogenesis 73, and Slit2/Robo4 can inhibit tumor angiogenesis 74, 75. To date, the research on the role of Slit/Robo signaling in angiogenesis is more focused on the direction of tumor development. However, few studies are related to bone development or metabolic bone balance. Whether Slit2 also plays a regulatory role in angiogenesis-osteogenesis coupling, such as Slit3 is still an interesting question worth exploring.

## Conclusion and Perspectives

Slit and its main receptor Robo were first found in the study of nerve development and are considered classical axon guide molecules. With the in-depth understanding of Slit/Robo signaling, its role in other fields, such as tumor development, angiogenesis, bone development, and inflammatory regulation, has been gradually discovered. In recent years, an increasing number of studies have shown that Slit is a novel bone coupling factor that can regulate bone resorption and bone formation at the same time. In addition, because of the angiogenic effect of Slit, H-type blood vessels are associated with bone metabolism via Slit. The application of Slit in the treatment of metabolic bone diseases is also being explored; for instance, the injection of truncated recombinant Slit3 in mice can significantly save bone loss.

However, numerous unanswered questions remain regarding the role of the Slit/Robo signal in bone metabolism. For example, is the main source of Slit3 in the skeletal microenvironment from osteoblasts or osteoclasts? Does Slit2 have any effect on osteoblast biology? The Slit/Robo signaling pathway has various downstream signals in different spatiotemporal environments, resulting in the complexity of Slit/Robo signals. This makes it more difficult to clarify the specific mechanism of Slit/Robo signaling in bone metabolism.

According to the current research results, Slit shows good application prospects and therapeutic potential in the field of bone metabolism. Therefore, it is necessary to further explore and clarify the molecular mechanism of Slit/Robo in regulating bone homeostasis, which is of great significance to improve the molecular regulatory network of bone metabolism and explain the contradictory results reported in various existing studies. Simultaneously, fully exploiting the therapeutic potential of Slit family proteins in metabolic bone diseases and improving their clinical applicability are promising future research directions.

## Abbreviations

BMP: Bone morphogenetic protein
CGRP: Calcitonin gene related peptide
CXCR4/CXCL12: C-X-C motif chemokine receptor 4/C-X-C motif chemokine ligand 12
RhoA: Ras homolog family member A
Rac: Ras-related C3 botulinum toxin substrate
UNC5B: Unc-5 Netrin Receptor B
LRR: Leucine-rich repeats
EGF: Epidermal growth factor
Ig: Immunoglobulin
FNⅢ: Fibronectin type Ⅲ
CC: Cytoplasmic tail
Dscam1: Down syndrome cell adhesion molecule 1
PKA: Protein kinase A
ADAM10: A Disintegrin and metalloproteinase domain-containing protein 10
HSPGs: Heparan sulfate proteoglycans
BMUs: Basic multicellular units
TRAP: Tartrate-resistant acid phosphatase
CTR: Calcitonin receptor
Cdc42: Cell division control protein 42 homolog
srGAP: Slit-Robo GTPase activating protein
Abl: Abelson
